# The Role of Neuropeptides in Pathogenesis of Dry Dye

**DOI:** 10.3390/jcm10184248

**Published:** 2021-09-19

**Authors:** Daniel Duck-Jin Hwang, Seok-Jae Lee, Jeong-Hun Kim, Sang-Mok Lee

**Affiliations:** 1 Department of Ophthalmology, HanGil Eye Hospital, Incheon 21388, Korea; hallelu7@gmail.com; 2 Department of Ophthalmology, College of Medicine, Catholic Kwandong University, Incheon 21388, Korea; 3 Fight against Angiogenesis-Related Blindness (FARB) Laboratory, Clinical Research Institute, Seoul National University Hospital, Seoul 03080, Korea; bonheur0809@gmail.com (S.-J.L.); steph25@snu.ac.kr (J.-H.K.); 4 Department of Biomedical Sciences, College of Medicine, Seoul National University, Seoul 03080, Korea; 5 Department of Ophthalmology, College of Medicine, Seoul National University, Seoul 03080, Korea; 6 Advanced Biomedical Research Center, Korea Research Institute of Bioscience & Biotechnology, Daejeon 34141, Korea

**Keywords:** dry eye, neuropeptide, pathogenesis, neurogenic inflammation, corneal nerve, substance P, calcitonin gene-related peptide, neuropeptide Y, vasoactive intestinal peptide

## Abstract

Neuropeptides are known as important mediators between the nervous and immune systems. Recently, the role of the corneal nerve in the pathogenesis of various ocular surface diseases, including dry eye disease, has been highlighted. Neuropeptides are thought to be important factors in the pathogenesis of dry eye disease, as suggested by the well-known role between the nervous and immune systems, and several recently published studies have elucidated the previously unknown pathogenic mechanisms involved in the role of the neuropeptides secreted from the corneal nerves in dry eye disease. Here, we reviewed the emerging concept of neurogenic inflammation as one of the pathogenic mechanisms of dry eye disease, the recent results of related studies, and the direction of future research.

## 1. Introduction

Systems that can resist mechanical damage and invasion of pathogens protect our bodies from possible loss of function, and the eyes are no exception [1]. In contrast to the anterior chamber, which is known to be immunologically privileged, the ocular surface is well equipped with defense systems and with systems for the constant surveillance of damage and pathogen entry, in which neurogenic inflammation plays an important role [2,3,4]. Damage or stimulation to sensory nerve endings caused by pathogens or mechanical damage results in the secretion of neuropeptides, which play important roles in triggering and regulating inflammatory responses, and this series of processes is called neurogenic inflammation [2,3]. Neurogenic inflammation is caused by small sensory nerves with relatively slow conduction speeds (unmyelinated C-fibers and finely myelinated A-fibers, Aδ fibers), which are densely innervated in the ocular surface [2,5]. Neuropeptides, including small-molecule peptides secreted by nerve endings represented by substance P (SP), calcitonin gene-related peptide (CGRP), vasoactive intestinal peptide (VIP), and neuropeptide Y (NPY), are known as important players in neuroimmune crosstalk [2,6]. Though neurogenic inflammation is important for wound healing and protection from infection [1,7], it is also regarded as an important pathogenic mechanism for some diseases, including allergic diseases, atopic dermatitis, psoriasis, rosacea, fibromyalgia, inflammatory bowel diseases, autoimmune enteritis, traumatic brain injury, pain, and migraine [6,8,9,10,11,12]. Dry eye disease (DED) is a common eye disease with a prevalence of 5%–50%, according to a recent meta-analysis of the published worldwide prevalence data [13]. In DED, possible pathogenic roles of neurogenic inflammation and neuropeptides have been previously suggested on the basis of the growing knowledge on the role of inflammation in DED and the presence of densely innervated sensory nerve endings in the ocular surface [2,14,15,16]. As studies supporting this idea have recently been published, this review presented a summary of recent findings on the role of neuropeptides in DED and suggested directions for future research.

## 2. The Role of the Immune and Nervous Systems in the Pathogenesis of Dry Eye Disease

### 2.1. Immunopathogenesis of Dry Eye Disease

DED is a common eye disease throughout the world, with a prevalence of 5%–50% reported, and age and female sex, among the various factors, are consistently associated with DED in meta-analysis [13,17]. In 1995, the National Eye Institute/Industry Workshop on Dry Eyes did not consider inflammation or the immune system to be important pathogenic mechanisms of DED except in Sjögren’s syndrome, as DED was considered a tear film disorder caused by tear deficiency or excessive tear evaporation [18,19]. However, since 1995, studies have reported an increase in proinflammatory cytokines, T-cell populations, chemokines, and their receptors even in non-Sjögren’s DED, and inflammation and the immune system, including both innate and adaptive immune responses, are currently recognized as important contributors to the pathogenesis of DED [15,19,20,21,22].

The current concept of immunopathogenesis of DED is summarized as follows [16,19,23]. Hyperosmolar stress caused by desiccating stress (DS) can hijack the physical barrier system through the activation of intracellular signaling pathways, mitogen-activated protein kinase (MAPK) and nuclear factor kappa-light-chain-enhancer of activated B cells (NFκB) stress signaling pathways and can initiate proinflammatory cytokine production [15,16,24,25,26]; these proinflammatory cytokines, including interleukin-1 (IL-1), tumor necrosis factor-α (TNF-α), and IL-6, released from stressed ocular-surface epithelium induce epithelial damage and promote the activation and maturation of resident immature antigen-presenting cells (APCs) and natural-killer (NK) cells [23,27]. In addition, this proinflammatory environment promotes the expression of adhesion molecules such as intercellular adhesion molecule-1 (ICAM-1) in the conjunctival and corneal epithelium and vascular endothelium, helping leukocytes migrate to the site of inflammation [28]. 

Mature APCs (mAPCs) activated by DS migrate to draining lymph nodes (LNs) via afferent lymphatic vessels, and this process can be promoted by vascular endothelial growth factor (VEGF)-C and VEGF-D [16,23,29,30]. In draining LNs, mAPCs induce the activation and expansion of naive T cells into effector T cells, interferon- γ (IFN-γ)-secreting CD4+ T cells (Th1), and IL-17-secreting CD4+ T cells (Th17) [31,32]. Th17 cells antagonize regulatory T cell (Treg) function, and the resulting dysfunctional Tregs that are unable to regulate further expansion of effector T cells, particularly Th17 cells, in draining LNs [33]. These uninhibited effector T cells migrate from draining LNs to the ocular surface via efferent blood vessels, which can be promoted by VEGF-A, under the influence of increased levels of chemokines on the ocular surface, including CCL3/4/5, CXCR9/10 (for Th1 influx), and CCL20 (for Th17 influx) [23,34]. Effector Th1-secreted IFN-γ and Th17-secreted IL-17 demonstrate their pathogenic effects by facilitating the production of proinflammatory cytokines, matrix metalloproteinases (MMP-3 and MMP-9), chemokines, cell adhesion molecules, and pro-lymphangiogenic molecules (VEGF-C and VEGF-D).

In this way, recruited effector T cells promote the infiltration of pathogenic immune cells, leading to further damage of the ocular surface with corneal barrier disruption and decreased conjunctival goblet cell density by IFN-γ and inducing corneal lymphangiogenesis by IL-17 [32,35]. This self-perpetuating inflammatory cycle is central to the pathogenesis of DED [16].

The experimental evidence for this immunopathogenesis theory of DED, based on T-cell-associated adaptive immunity, is based on animal studies using adoptive transfer and studies on memory Th17 cells [36,37,38]. T cell-mediated inflammation of the cornea, conjunctiva, and lacrimal glands of recipient mice not previously exposed to DS was induced by adoptive transfer of CD4+ T cells from mice primed with DS, and this inflammation was reversed by anti-CD4 treatment [36]. In a chronic DED murine model, a significant population of memory T cells, primarily Th17 cells, was recovered and adoptive transfer of these cells led to more severe and rapidly progressing DS-induced DED compared with the control [39]. Increased IL-23 and decreased IL-2 may contribute to the generation of these memory Th17 cells, and IL-7 and IL-15 have been shown to play important roles in the maintenance of memory Th17 cells [37,40,41]. 

DED involves a unique form of pathological angiogenesis characterized by ‘lymphangiogenesis without associated angiogenesis’, which has been observed in experimental and clinical settings [16]. The presence of monocytic cells stained with the lymphatic endothelium-specific marker, lymphatic vessel endothelial hyaluronan receptor 1 (LYVE-1) in the conjunctiva has been described and is associated with the growth of lymphatic vessels into the cornea [42]. Immunohistochemical analysis of dry eye corneas confirmed the infiltration of LYVE-1-expressing macrophages and lymphatic vessels [43]. Dry eye induction increases the expression of factors that promote lymphangiogenesis, including VEGF-C and VEGF-D and the related receptors VEGFR-2 and VEGFR-3 [16]. VEGF-A is also upregulated and contributes to lymphangiogenesis through the recruitment of VEGF-C- and VEGF-D-producing macrophages. The identification of newly formed lymphatic vessels in the cornea provides a potential pathway by which antigens and mAPCs migrate from the ocular surface to draining LNs [16,23].

### 2.2. Role of Sensory Nerves in the Pathogenesis of Dry Eye Disease

The abundant supply of sensory and autonomic nerve fibers on the ocular surface plays an important role in maintaining healthy epithelia and serves as the main source of neurogenic inflammation [2]. Along with stromal keratocytes, the subbasal nerve plexus secretes a number of neuropeptides and neurotrophic factors, such as nerve growth factor (NGF), that promote cell mitogenesis and migration, DNA synthesis, neurite extension and survival, keratocyte proliferation, and regulation of epithelial stem cells, resulting in epithelial growth, proliferation, differentiation, and production of collagen type VII [44,45,46,47]. Ocular surface epithelial cells seem to produce the soluble factors NGF and glial cell-derived neurotrophic factor (GDNF), causing a neurotrophic effect. 

In DED-induced corneas, decreased density and altered morphologic structure of the subbasal nerves have been reported [21,22,47,48,49,50,51,52,53]. These abnormalities are generally found to correlate with DED severity. Tear hyperosmolarity-mediated epithelial damage or hyperosmolarity itself can expose corneal nerve endings to mechanical and inflammatory insults [16,54,55,56]. The reduction in corneal sensitivity in DED promotes neurogenic stress, contributing to impairment of ocular surface homeostasis [48,57,58]. Additionally, inflammatory cytokines released in turn increase the synthesis of neurotrophic factors that stimulate nerve growth, potentially explaining the altered nerve morphologic structures including nerve sprouts, tortuosity, and thinning commonly observed in DED [59]. Corneal nerve abnormalities lead to further ocular surface damage and help perpetuate the vicious inflammatory cycle of DED [51]. 

The concept of the lacrimal functional unit was presented in 1998, and this unit is composed of lacrimal glands (both main and accessory), the ocular surface, and interconnecting innervation [20,60]. The afferent branch of the loop includes stimulation of free nerve endings enriched in the cornea, traveling of impulses within trigeminal neuronal cells to the mid-brain (pons) where they synapse, integration of the signal with cortical and other neural input, and the efferent branch of the loop sends fibers through the pterygopalatine ganglion and then to the main and accessory lacrimal glands (Wolfring and Krause) [20]. The importance of the roles of innervation in the regulation of tear secretion is highlighted in this concept and neurosensory dysfunction has been reported to play an important role in the pathophysiology of DED [52,61]. 

The induction of neurogenic inflammation within the ocular surface and lacrimal glands through the release of neuropeptides has been suggested to be a contributor to the pathogenesis of DED [20,21,62]. An inflamed lacrimal gland may produce ‘toxic tears’ containing proinflammatory cytokines, disrupting ocular surface homeostasis and exacerbating the innate inflammatory response [6,63,64]. SP, CGRP, VIP, and NPY released from ocular surface epithelial cells, lacrimal gland tissues, and nerve endings at inflammatory sites seem to modulate the infiltration and activation of immune cells, causing reflex tearing and ocular discomfort [65,66]. Neuromediators are involved in chronic ocular surface diseases such as dry eye, with SP and CGRP promoting local inflammation by inducing vasodilatation, extravasation of leucocytes, activation of immune cells, and release of several cytokines [67,68]. VIP and NPY have anti-inflammatory properties manifesting as inhibited proliferation of T cells and Th1 response and modulated release of cytokines, chemokines, and nitric oxide [69,70].

Recently, the role of cold thermoreceptor neurons expressing transient receptor potential cation channel subfamily M member 8 (TRPM8) has been highlighted as an important regulatory function for tear secretion and eye blinking [71,72,73,74], mediated by sensing cooling, hyperosmolarity, and wetness of the corneal surface [74,75,76]. Upregulation of TRPM8 receptors after surgical injury of corneal nerve fibers was suggested to be a mechanism of increased tear production and unpleasant dry eye sensation [77,78]. This observation may more rationally explain the process by which hyperosmolar stress hijacks the physical barrier system of the ocular surface and induces inflammation by suggesting an additional pathway through the activation of the TRPM8 cold thermoreceptor, which induces the aforementioned MAPK-NFκB intracellular stress signaling pathway.

Tear hyperosmolarity has been shown to activate not only cold thermoreceptor neurons but also high-threshold cold sensitive plus dry-sensitive (HT-CS + DS) nociceptive neurons in mice and rat corneas [55,56,75]. Activation of these neurons was followed by depressed or even completely abolished responses to dryness and morphological abnormalities in subbasal nerves that appeared to be undergoing degeneration in the rat cornea [54]. By electrophysiological studies of single neurons of the trigeminal ganglion innervating the rat cornea, it was reported that spontaneous blinking can be controlled by tear osmolality and ocular surface cooling [79]. Recently, in a murine model of tear hyperosmolarity, immunopathogenic responses similar to DED were induced without DS and the effect of adoptive transfer of T cells was also shown, suggesting that tear hyperosmolarity acts as a critical factor in DED induction by DS [56].

## 3. Role of Neuropeptides and Its Distribution in Ocular Surface and Lacrimal Gland

### 3.1. Representative Neuropeptides and Their Receptors

Neuropeptides released by afferent nerve terminals function as both neurotransmitters and neuromodulators that lead to pain sensation [80,81,82] or neurogenic inflammatory responses [3,5,6]. The representative neuropeptides known to modulate immune reactions in response to external stimuli (mechanical, cold, and chemical stimuli; inflammatory mediators; damage-associated molecular patterns; and pathogen-associated molecular patterns) include SP, CGRP, VIP, and NPY [82,83,84,85,86,87].

SP is an 11-amino acid neuropeptide in the tachykinin family that is mainly secreted by neuronal cells and inflammatory cells [88,89]. The SP precursor is encoded by the preprotachykinin-A (TAC-1) gene and is cleaved into active undecapeptide by alternative splicing, resulting in a biologically active fragment [90]. The bioactivity of SP is regulated by the activation of three tachykinin receptors, named neurokinin-1 receptor (NK1R), NK2R, and NK3R, which belong to the superfamily of rhodopsin-like G protein-coupled receptors, and they have a seven-transmembrane domain [91]. SP preferentially binds to NK1R and exerts numerous physiological and pathological activities through its high affinity for NK1R [92,93,94]. The binding of SP and NK1R triggers phospholipase C-dependent processes, adenylate cyclase, and Akt/protein kinase B, which promotes DNA synthesis, cell proliferation/survival/motility, vasodilation, pain transmission, and endocrine/paracrine secretion [95]. Recently, it has been reported that SP acts as an endogenous agonist of the mast cell-specific receptor Mrgprb2, independent of NK1R, inducing mast cell activation, which can lead to neurogenic inflammation [96].

CGRP is a multifunctional neuropeptide composed of 37 amino acids with an N-terminal disulfide bond and amidated C-terminus. CGRP exists in two isoforms, α and β, which are expressed by CALCA and CALCB, respectively [97]. Both peptides are differentially regulated and expressed but have nearly indistinguishable activities [98]. The CGRP receptor is a G protein-coupled receptor complex that contains three subunits: calcitonin receptor-like receptor (CLR), receptor activity-modifying protein 1 (RAMP1), and receptor component protein (RCP) [99,100]. CGRP binds to the heterodimer of CLR and RAMP1, which are transmembrane proteins, and RCP facilitates coupling of the G protein alpha s subunit (Gαs) [101]. Furthermore, CGRP can also bind to receptors of two peptides in the calcitonin family, adrenomedullin and amylin, which are composed of CLR and RAMP2 or RAMP3, and the calcitonin receptor and RAMP1, respectively [102]. Traditionally, CGRP receptors have been classified into CGRP1 and CGRP2, which have different sensitivities; however, because of a wide range of affinities of the antagonist for the receptor (pA_2_) that differs by tissue, heterogeneous receptors have been suggested to respond to CGRP in tissues [102]. The CGRP receptor is coupled to Gαs proteins, leading to activation of the cyclic adenosine monophosphate (cAMP)-protein kinase A signaling pathway and triggering activation of phospholipase C, elevation of intracellular calcium levels, and activation of protein kinase C [103].

VIP is synthesized as an active peptide with 28 amino acids from the precursor molecule, and the gene encoding this peptide resides on chromosome 6 [104,105]. VIP is primarily expressed in parasympathetic nerve terminals originating from the central nervous system. VIP and pituitary adenylate cyclase-activating polypeptide (PACAP) are two highly related neuropeptides, and there are three types of G protein-coupled receptors that bind to VIP and PACAP: VIP/PACAP receptor 1 (VPAC_1_R), VPAC_2_R, and PACAP receptor 1 (PAC_1_R) [106]. VPAC_1_R and VPAC_2_R bind to VIP with equal affinity, whereas PAC_1_R binds to VIP with much lower affinity [100,107]. The interaction between VIP and G protein-coupled receptors activates the heterotrimeric G protein Gs, leading to elevation of cAMP, a secondary messenger that advances the cell signaling cascade progression [108].

NPY has a 36-amino acid structure with tyrosine residues at the ends [109,110] and is predominantly found in sympathetic neurons [111]. NPY receptor subtypes have been identified as G protein-coupled receptors that have been designated “Y” receptors (Y_1_, Y_2_, Y_3_, Y_4_, Y_5_, and Y_6_). Among these receptors, five receptors have been cloned from mammals, Y_1_, Y_2_, Y_4_, Y_5_, and Y_6,_ but only four of the receptors are functional in humans (hY_1_, hY_2_, hY_4_, and hY_5_) [100,112]. These receptors generally induce signaling by coupling to Gi or Go proteins, which leads to the inhibition of adenylyl cyclase [113]. In addition, NPY receptor-related signaling can stimulate phospholipase C, resulting in the accumulation of inositol 1,4,5-trisphosphate (IP_3_) and modulation of calcium and potassium channels [114].

### 3.2. Role of Neuropeptides in Neurogenic Inflammation

#### 3.2.1. Concept of Neurogenic Inflammation 

Neurogenic inflammation is the inflammation caused by sensory and autonomic nerve terminal-derived substances, including neuropeptides, which are released in response to nerve damage or stimulation [2,115,116]. Neuropeptides lead to leukocyte influx and chemotaxis of immune cells to the inflammation site by inducing vasodilation/plasma extravasation and edema and stimulating the release of chemotactic chemokines. Furthermore, recruited leukocytes such as monocytes and neutrophils release inflammatory mediators to stimulate innate/adaptive immune responses [2,68,117]. This inflammatory process is generally thought to be controlled by crosstalk between nerves and immune cells, such as mast cells, macrophages, eosinophils, and lymphocytes. However, severe nerve injuries that disrupt the balance between the nervous and immune systems result in excessive or prolonged release of neuropeptides at sites of inflammation. These neuropeptides promote inflammation by recruiting and activating innate immune cells (dendrite cells and mast cells) and adaptive immune cells (T lymphocytes) by binding the expressed cell membrane receptors of neuropeptides [118,119,120,121], which subsequently induces the release of inflammatory cytokines such as TNF-α, IL-1α, IL-1β, IL-6, IL-8, and IFN-γ [4,16,89], ultimately leading to tissue damage and loss of function. Interestingly, because immune cells synthesize neuropeptides during inflammation and because sensory nerves also express receptors of proinflammatory cytokines, the action of the neuroimmune system is bidirectional [122,123,124,125]. When this bidirectional reaction is poorly controlled, neuropeptides are released in excess or for a prolonged time due to a vicious cycle, and the resulting neurogenic inflammation acts as a pathogenic mechanism of various diseases, such as allergic diseases, atopic dermatitis, psoriasis, rosacea, fibromyalgia, inflammatory bowel diseases, autoimmune enteritis, traumatic brain injury, pain, and migraine [6,8,9,10,11,12].

#### 3.2.2. Role of Substance P (SP) in Neurogenic Inflammation

SP is involved in numerous physiological and pathological activities, such as nociception [31,63,64], inflammation [65], hemangiogenesis and lymphangiogenesis [66,67,68], and wound healing [69,70]. Notably, SP has been reported to be a key molecule in neurogenic inflammation. NK1R, a receptor with high affinity for SP, is expressed not only in peripheral neurons, vascular endothelial cells, and lymphatic endothelial cells, but also in many inflammatory cells, such as dendritic cells, macrophages, mast cells and lymphocytes [68]. SP/NK1R-mediated activation stimulates chemotactic properties in inflammatory cells by releasing chemokines such as CCL2, CCL4, CXCL2, and IL-8 [126,127,128] and promotes the production of proinflammatory cytokines such as TNF-α, IL-1β, IL-6, and IFN-γ [128,129]. 

Furthermore, the SP/NK1R system modulates the activation of innate and adaptive immune systems. SP upregulates cytotoxicity-associated molecules, perforin and granzyme, to enhance the cytotoxicity of human natural-killer cells [130], enhances phagocytosis of macrophages [131], and participates in promoting the survival and maturation of dendritic cells [132]. The SP/NK1R interaction inhibits IL-10 secretion by dendritic cells and promotes the generation of T helper 17 (Th17) lymphocytes from memory CD4+ T cells [133]. SP also plays an important role in adaptive immunity through activation of T cells. SP enhances IL-2 expression in human T cells and promotes T cell proliferation [134,135]. Binding of SP to NK1R in T cells also induces the production of IFN-γ and promotes optimal Ca^2+^ flux [136,137], maintaining T cell survival and efficient Th1- and Th17-mediated immunity [137]. Taken together, SP induces proinflammatory activity in both innate and adaptive immunity.

#### 3.2.3. Role of Calcitonin Gene-Related Peptide (CGRP) in Neurogenic Inflammation

CGRP is a neuropeptide that is stored in sensory neurons and is widely expressed in the central and peripheral nervous systems. CGRP is a potent vasodilatory component of neurogenic inflammation that mediates pain transmission [138]. CLR and RAMP1, which are CGRP receptors, are found on dendritic cells [139], macrophages [140], mast cells [141], and T/B lymphocytes [142], as well as on nerve fibers. 

CGRP acts as a key regulator of lymphocyte differentiation and cytokine production. The interaction of T cells with APCs, such as dendritic cells and macrophages, is influenced by CGRP expression. Studies have demonstrated that CGRP leads to Th2 polarization by influencing T cell differentiation [143,144,145] and enhancing the production of Th2 cytokines such as IL-4, CCL17, and CCL22 while decreasing the Th1 cytokines IFN-γ and IL2 [142,144,146]. In relation with Th1-type immune responses, CGRP mainly exerts anti-inflammatory and immunosuppressive effects, which inhibit the production of proinflammatory cytokines and diminish the capacity of immune cells to present antigen to T cells [144,147,148,149]. The upregulation of IL-10, an immunomodulatory cytokine; induction of inducible cAMP early repressor, a transcription repressor; and inhibition of NF-κB activity are also induced by CGRP [149,150]. On the other hand, CGRP has been shown to be proinflammatory in several diseases mainly associated with Th2-type immune responses, including allergic airway disease, atopic dermatitis, autoimmune encephalomyelitis, migraine, and obstructive nephritis [141,142,151,152,153,154].

Therefore, CGRP exhibits either a proinflammatory or an anti-inflammatory depending on the disease situation, which has been debated with evidence of a range of proinflammatory and anti-inflammatory conditions.

#### 3.2.4. Role of Vasoactive Intestinal Polypeptide (VIP) and Pituitary Adenylate Cyclase-Activating Polypeptide (PACAP) in Neurogenic Inflammation

VIP is primarily expressed in parasympathetic nerve terminals with wide distribution in the body. VIP is typically involved in gastrointestinal epithelial secretion, smooth muscle relaxation, and vasodilation [155].

Regarding the neurogenic inflammation process, VIP has immunomodulatory properties contributing to the regulation of immune deviation and the control of acute inflammation in peripheral organs [156,157]. In addition to neurons, VIP is highly synthesized in immune cells such as macrophages, dendritic cells, and T lymphocytes, which also express VIP receptors. VIP promotes the Th2-type immune response in macrophages and dendritic cells by inducing the Th2 cytokines IL4 and IL5, whereas VIP inhibits Th1-type immune responses by downregulating IFN-γ, IL-2, TNF-α, proinflammatory chemokines, and Toll-like receptor expression, resulting in an increased ratio of Th2/Th1 cells [158,159]. Furthermore, VIP not only promotes the generation of regulatory T cells that subsequently suppress the activation of autoreactive T cells by producing IL-10 and TGF-β, but also dampens the costimulatory activity of APCs by suppressing the expression of costimulatory molecules such as major histocompatibility complex-II, CD80, CD86, and proinflammatory chemokines [160]. Therefore, VIP contributes to the maintenance of an anti-inflammatory effect and controls immune tolerance in an environment of neurogenic inflammation. 

PACAP is a 27- or 38-amino acid neuropeptide that belongs to the secretin/glucagon/VIP family. PACAP shows high homology to VIP and shares three VIP G protein-coupled receptors, VPAC_1_R and VPAC_2_R, and PAC_1_R. Regarding neurogenic inflammation, PACAP exerts an immunosuppressive and neuroprotective effect in response to inflammation or injury in the nervous system [161,162]. 

#### 3.2.5. Role of Neuropeptide Y (NPY) in Neurogenic Inflammation

NPY is a potent stimulant of food intake to restore homeostatic conditions and affects vascular and gastrointestinal smooth muscle function by binding to the Y1 receptor [163]. NPY also contributes to the immune response by activating immune cells that express NPY receptors. In the immune system, NPY is mainly derived from sympathetic nerves acting on immune organs [164]. NPY stimulates leukocyte adhesion and migration and induces the activation of monocytes and macrophages to produce pro- and anti-inflammatory molecules such as IL-4, IL-6, IL-12, IL-1β, TNF-α, and TGF-β [165,166]. Similar to CGRP and VIP, NPY induces APCs to migrate and recruits them to inflammatory sites, and it mainly exerts anti-inflammatory effects by promoting Th2 polarization through upregulation of the Th2-polarizing cytokines IL-6 and IL-10 in APCs [167]. In addition, NPY promotes immune tolerance activity in monocytes and T cells by mediating the activation of tolerogenic APCs and regulatory T cells, which act as cellular negative regulators of inflammation [168,169,170].

#### 3.2.6. Role of Other Neuropeptides in Neurogenic Inflammation

Other neuropeptides, such as alpha-melanocyte-stimulating hormone (α-MSH) and galanin, are also released from sensory nerve terminals and contribute to the process of neurogenic inflammation. α-MSH is a 13-amino acid neuropeptide and is generated from a precursor protein called proopiomelanocortin through posttranslational proteolytic cleavage. In several in vitro and in vivo studies, α-MSH has been shown to exert potent anti-inflammatory properties by suppressing lymphocyte proliferation; suppressing the proinflammatory effects of IL-1, IL-6, TNF-α, IFN-γ, and NFkB; and promoting the secretion of IL-10 [171,172]. α-MSH also induces regulatory activity in T cells, which involves the conversion of effector T cells into regulatory T cells [173].

Galanin is a 29-amino acid neuropeptide processed from a 123-amino acid precursor molecule, preprogalanin, that exerts multiple biological effects following the binding of three galanin-receptor subtypes, galanin receptor 1 (GalR1), GalR2, and GalR3 [174]. Studies have reported that galanin inhibits plasma extravasation and the release of proinflammatory cytokines in experimental inflammatory diseases in vivo such as dermatitis, synovitis, and colitis [175,176,177,178]. Galanin also has an immunosuppressive effect, decreasing the population of active CD8+ T cells and Th17 cells and modulating the expression of chemokines and anti-inflammatory cytokines [179,180].

### 3.3. Distribution of Nerves and Expression of Neuropeptides in Ocular Surface and Lacrimal Gland

#### 3.3.1. Sensory and Autonomic Nervous Systems of Eye

**Figure 1:**
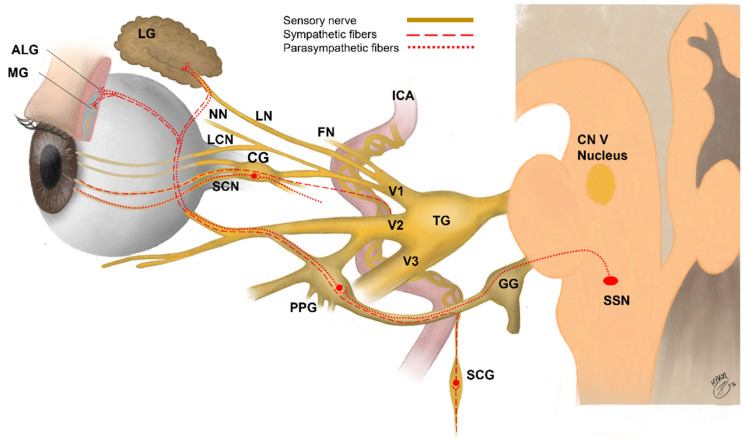
Schematic drawing for innervation of the lacrimal functional unit ALG: accessory lacrimal glands, CG: ciliary ganglion, CN: cranial nerve, FN: frontal nerve, GG: geniculate ganglion, ICA: internal carotid artery, LCN: long ciliary nerve, LG: lacrimal gland, LN: lacrimal nerve, MG: Meibomian gland, NN: nasociliary nerve, SCG: superior cervical ganglion, SCN: short ciliary nerve, SSN: superior salivatory nucleus, PPG: pterygopalatine (sphenopalatine) ganglion, TG: trigeminal ganglion (modified from Netter, FH, The Ciba Collection of Medical Illustrations, CIBA-Geigy Corporation, 1991).

Sensory innervation of the eye consists mainly of primary sensory neurons of the trigeminal ganglion (TG) (Figure 1, Table 1). Sensory nerve fibers (mechano-nociceptors, Aδ fibers; cold receptors, Aδ and C fibers; and polymodal nociceptors, Aδ and C fibers) supplying the ocular surface have cell bodies in the ophthalmic and maxillary regions of the TG [46,181,182,183]. Sensory nerves to the cornea and the bulbar conjunctiva connect to the eye initially via the nasociliary branch of the ophthalmic nerve, and then via the long ciliary nerves and the communicating branch to the ciliary ganglion [73]. Sympathetic fibers originate from the superior cervical ganglion, and parasympathetic fibers originate from pterygopalatine ganglion and are considered to travel together to the ocular surface in humans (Table 1) [46,184]. The ciliary ganglion gives rise to short ciliary nerves, which contain both sensory and autonomic fibers. The long and short ciliary nerves pass the sclera at the back of the eye and run forward to the anterior segment in the suprachoroidal space [73,185]. The ciliary nerves separate to form multiple branches that reach the corneal limbus at the same intervals around its circumference, forming a circumferentially arranged pericorneal plexus [73,186]. The sensory and autonomic nerve fibers supplying the cornea and limbal conjunctiva exit anteriorly from this plexus. The branches of the ophthalmic nerve and branch of the maxillary nerve innervate in the remaining bulbar conjunctiva, entire palpebral conjunctiva, and skin covering the eyelid margins [73,187,188].

#### 3.3.2. Sensory and Autonomic Innervation of Cornea

The cornea has the densest innervation within the human body [193]. Most of its nerves, derived from the TG, are sensory, although a small contribution of sympathetic nervous system from the superior cervical ganglion and the parasympathetic nervous system has also been reported [46]. In animals, approximately 20%–30% of the axons supplying the cornea are thinly myelinated and the remainder are unmyelinated [194,195]. However, myelinated fibers lose their myelin sheath within approximately one millimeter of entering the corneal stroma [196]. Immediately after entering the cornea, the nerve bundles anastomose to neighboring bundles to form the stromal plexus concentrated in the anterior one-third of the stroma [197]. The most superficial layer of the stromal nerve plexus, located just below Bowman’s layer, is known as the subepithelial plexus [197]. Most axons entering the corneal stroma penetrate Bowman’s layer from the subepithelial plexus at an acute angle and terminate as unencapsulated nerve endings in the corneal epithelium [196]. Human subbasal nerve fibers contain axons that lose their Schwann cell envelope when entering the epithelium [196,198], and the subbasal nerve plexus constitutes the densest layer of human corneal innervation [73]. CGRP and SP are expressed by 15%–41% and 3%–31% of corneal nerve fibers, respectively [85,199]. CGRP and SP in nerve fibers are largely distributed throughout the anterior corneal stroma and the epithelium, forming a highly branched subepithelial plexus, whereas there are far fewer NPY and VIP fibers, and they are mainly distributed in the anterior stroma, rarely branching and rarely seen below basal epithelial cells (Table 2). Jones and Marfurt reported denervation studies showing that CGRP and SP nerve fibers originate from sensory nerves, whereas VIP fibers originate from parasympathetic nerves, and NPY originate mainly from sympathetic nerves but partly from parasympathetic nerves [200].

#### 3.3.3. Sensory and Autonomic Innervation of Conjunctiva and Eyelid Margin

Much less is known about the sensory innervation of the conjunctiva and the margins of the eyelids than that of the cornea [73]. The conjunctiva is innervated by unmyelinated sensory, sympathetic, and parasympathetic nerve fibers originating from the trigeminal, superior cervical, and pterygopalatine ganglion, respectively [189,214]. In the conjunctiva, the peptidergic nerve endings are located mostly around the blood vessels of the stroma but can also be found less frequently in the epithelium or around the acini of meibomian glands and lymph follicles (Table 2) [73,206,215]. CGRP-immunoreactive fibers innervate the epithelium, substantia propria, and stroma of the conjunctiva in rats [207]. In particular, nearly 90% of these fibers innervate the palpebral conjunctiva with more fibers innervating closer to the mucocutaneous junction. Nerve fibers in the bulbar conjunctiva mainly innervate the vessels [3,207]. In the epithelium, the number of SP-immunoreactive nerve fibers is one-half that of CGRP-immunoreactive fibers, whereas the SP- and CGRP-secreting fibers encircling stromal blood vessels are similarly distributed [207,216]. NPY-immunoreactive fibers have been described as being in close proximity to conjunctival blood vessels and glands in rats [206]. VIP has shown an immunoreactive function along the epithelial-stromal junction and adjacent to goblet cells, where it stimulates mucin secretion from conjunctival goblet cells [208].

In the eyelid margin, the morphology of sensory nerve endings is more diverse and includes abundant Meissner corpuscles, Merkel disc endings, and free nerve endings [190]. These nerve endings are specialized for the detection of very low intensity mechanical stimuli [73].

#### 3.3.4. Sensory and Autonomic Innervation of Lacrimal Gland

The lacrimal gland is largely innervated by the parasympathetic (pterygopalatine ganglion), sympathetic (superior cervical ganglion), and sensory (trigeminal ganglion) nervous systems (Table 1) [191,213]. Autonomic nerves regulate the secretory activity of the lacrimal gland. The activity of these efferent nerves is regulated by reflexes initiated by activation of sensory neurons at the ocular surface. The lacrimal gland is innervated by both sympathetic and parasympathetic nerves, with the latter the more extensively innervated [73]. While nerve fibers in the human lacrimal gland predominantly express VIP and little CGRP or NPY, nerve fibers associated with interlobular blood vessels are mainly CGRP-positive and NPY-positive and rarely VIP-positive (Table 2). In addition, CGRP is expressed in the human epithelium of the interlobular and excretory ducts [212]. The NPY immunoreactivity distributes similarly to norepinephrine-containing neurons and is considered to have a mild stimulatory effect on protein secretion [217]. In humans, VPAC_1_R was identified on the basolateral membrane of acinar cells, whereas VPAC_2_R was found to be localized to myoepithelial cells [73]. 

#### 3.3.5. Sensory and Autonomic Innervation of Meibomian Gland

The neurobiology of meibomian glands was reviewed by Cox et al. in 2014 [192]. The nerve fibers from the sensory (trigeminal ganglion), parasympathetic (pterygopalatine ganglion), and sympathetic (cervical ganglion) nervous systems innervate meibomian glands [192]. While CGRP and VIP fibers have been described to be close to the acini of the meibomian glands in humans, CGRP is denser in the lid margin conjunctiva (Table 2) [62,192]. SP immunoreactivity is rare in meibomian glands in humans and is associated with a greater extent with their vasculature, whereas NPY nerve fibers are found around blood vessels in animal models [192].

## 4. Recent Results Showing the Roles of Neuropeptides in the Pathogenesis of Dry Eye Disease

### 4.1. Preclinical Study

**Figure 2:**
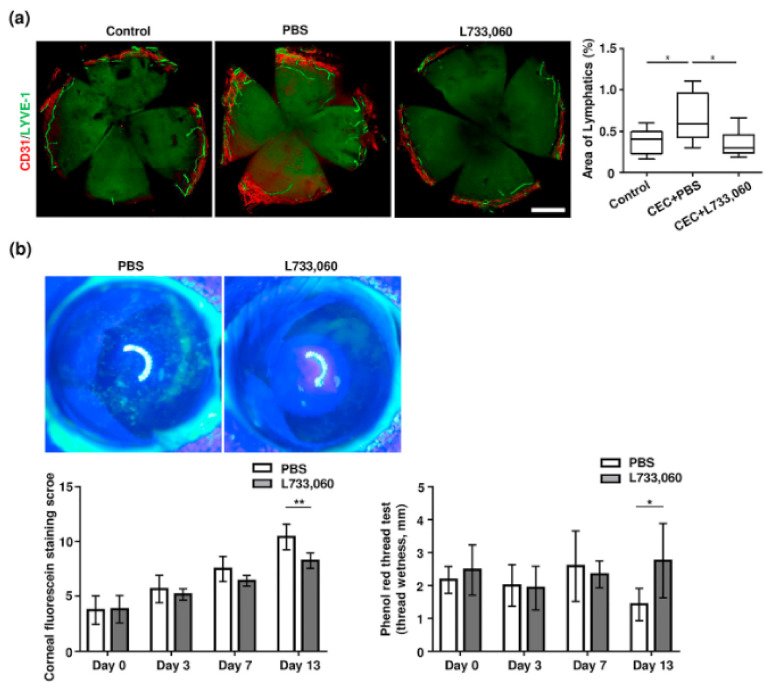
NK1R blockade ameliorates pathological corneal lymphangiogenesis and improves clinical signs of dry eye in vivo. (a) Representative immunofluorescence images of whole-mount cornea stained with CD31 (red) and LYVE-1 (green) and quantification of the percentage of lymphatics area covered with LYVE-1 in the cornea tissue at 14 days after induction of dry eye in CEC. NK1R antagonist (L733,060, 6.6 µg/5 µL) for the DED treatment group or same volume of PBS for PBS group was administered through subconjunctival injection every 48 h to 72 h from day 0 to day 14 of DED induction by CEC. Each value represents the mean ± SEM of nine independent whole-mount cornea. * *p* < 0.05 by Mann-Whitney U test. Scale bar = 500 μm. (b) Representative corneal fluorescein staining images showing punctate epithelial erosion at 14 days after induction of dry eye in CEC. Quantification of corneal fluorescein staining scoring according to the standard National Eye Institute grading system (Bethesda, MD) and the amount of tears measured by the phenol red thread test. * *p* < 0.05, ** *p* < 0.01 by Mann-Whitney U test. *n* = 6. CEC: controlled environmental chamber, LYVE-1: lymphatic vessel endothelial hyaluronan receptor 1, NK1R: neurokinin-1 receptor, PBS: phosphate-buffered saline [221].

Recently, several preclinical studies have reported using a dry eye animal model to elucidate the roles of neuropeptide/neuropeptide-specific receptors in the pathogenesis of DED (Table 3). There have been reports that the expression of SP is elevated in the cornea, conjunctiva, and trigeminal ganglion by exposure to DS in the controlled-environment chamber (CEC) [218,219,220]. Yu et al. [218] investigated the expression of SP at the ocular surface and evaluated its effect on the maturation of APCs, the key cells involved in the induction of the Th17-mediated response in DED. Topical treatment with NK1R antagonists has been shown to ameliorate DED severity through suppression of the Th17-mediated response [218,220]. Taketani et al. [220] showed that a significant increase in SP levels promotes Treg dysfunction in DED, and blockade of SP effectively restores Treg function and alleviates DED severity. Additionally, Liu et al. [219] investigated the regulation of angiogenesis by sensory nerves in response to DS using a coculture system of vascular endothelial cells and trigeminal ganglion sensory neurons harvested from DED mice. SP potently promoted the activation of the vascular endothelial cells in vitro, and blockade of SP signaling with spantide I, an antagonist of NK1R, significantly reduced corneal neovascularization in vivo, suggesting that sensory neurons directly promote angiogenesis via SP signaling in response to inflammation in the cornea. Furthermore, our group investigated the direct relationship between the SP/NK1R system and corneal lymphangiogenesis in DED using cultivated human lymphatic endothelial cells and a CEC-induced DED mouse model. We demonstrated that the SP/NK1R system promotes pathologic corneal lymphangiogenesis by controlling the expression of VEGFR3 and that blockade of the SP/NK1R system with L733,060, an antagonist of NK1R, significantly ameliorated pathological corneal lymphangiogenesis and improved the clinical signs of DED (Figure 2) [221].

In contrast, one study using thrombospondin-1-deficient mice, an aqueous-deficient dry-eye model of Sjögren’s syndrome, found that CGRP levels were substantially reduced but the SP level remained unchanged in the cornea [222]. It has also been reported that SP facilitates the healing of DS-induced corneal damage by inhibiting hyperosmotic stress-induced apoptosis of corneal epithelial cells and by directly inducing tissue-repairing M2-like macrophages [131,225]. These findings imply that blockade of NK1R can be more helpful in the early stage of DED before the appearance of punctate epitheliopathy.

Development of dry eye-like symptoms such as reduced tear secretion and corneal keratinization has been reported in PACAP-null mice, which could be improved by PACAP eye drops via the PAC_1_R/adenylyl cyclase/cAMP/protein kinase A (PKA)/aquaporin 5 (AQP5) cascade [224]. Some studies have demonstrated that administration of α-MSH ameliorates ocular surface dysfunction and dry eye symptoms by decreasing proinflammatory cytokine levels through activating the PKA-extracellular signal–regulated kinase (ERK) pathway [223,226,227].

The results from the preclinical studies to date can be summarized as follows. The corneal nerve distress caused by hyperosmolar stress in DED can cause increased SP levels in the ocular surface, which is possibly mediated by cold thermoreceptor neurons or HT-CS + DS nociceptive neurons that contribute to the maturation of resident APCs [218] following priming naive T cells to Th17 cells, but inhibiting the differentiation of Tregs [218,220]. The increased SP levels also cause lymphangiogenesis in the cornea by increasing the expression of VEGFR3 [221]. All these effects can be effectively suppressed by antagonizing NK1R [218,220,221]. In addition, there have been some reports for neuropeptide-related studies using DED animal models of an effect of α-MSH, which can reduce proinflammatory cytokines, and PACAP, which can stimulate tear secretion [223,224].

### 4.2. Clinical Study

#### 4.2.1. Dry Eye Disease

In DED patients, structural changes of the subbasal corneal nerve plexus have been reported in several clinical studies, specifically reduced subbasal corneal nerves, altered forms of corneal nerves, and decreased corneal sensitivity [48,51,52,53,228]. A possible role for neurogenic inflammation related to ocular surface dysfunction is suggested by the proinflammatory cytokines observed in tears and the altered levels of neuropeptides found in salivary glands of patients with Sjögren’s syndrome [20,229,230,231,232].

However, there have been very few clinical studies investigating the role of neuropeptides in DED. Lambiase et al. compared neuropeptides in tear fluid in 19 DED patients (Sjögren’s DED, *n* = 5 patients; non-Sjögren’s DED, *n* = 10 patients; and ocular cicatricial pemphigoid, *n* = 4 patients) and 12 controls and reported decreased CGRP and NPY levels and increased NGF levels in the DED patients [233]. In this study, CGRP levels in tears were significantly decreased, particularly in patients with non-Sjögren’s DED and ocular cicatricial pemphigoid. Reduced CGRP levels were inversely associated with disease severity and directly correlated with Schirmer test values [233]. It was assumed that the change in lacrimal gland function contributes to the decrease in CGRP levels in tears [233]. NPY levels in tears were also reduced and inversely correlated with the severity of DED [233], probably because of the anti-inflammatory properties of NPY. However, it is not known whether the reduced levels of NPY are a cause or consequence of DED [3] or whether the changes in tear NPY levels reflect changes in the lacrimal gland or arise as a consequence of ocular surface damage [73]. Interestingly, there were no significant changes in tear levels of SP and VIP in the aforementioned study [233], contrary to recent studies on SP using animal models. However, tear level of SP was reported to be decreased in patients with corneal hypesthesia or diabetic neuropathy [234,235,236] and to be correlated with a decreased nerve-fiber width in the subbasal nerve plexus and an increased tear concentration of IL-6 [237,238]. The lack of change in VIP with DED may reflect the very limited innervation of the cornea by VIP-expressing parasympathetic nerves [239].

#### 4.2.2. Dry Eye after Refractive Surgery and Neuropeptides

Refractive surgery results in a decrease in tear production, tear film quality, and a blinking reflex, which are involved in the pathogenesis of DED [240]. Changes in various neuropeptides have been observed following laser-assisted in situ keratomileusis (LASIK) or photorefractive keratectomy (PRK) [240,241,242,243,244]. Several studies have demonstrated increased SP levels in tears in the early post-LASIK period of up to 3 months [241,243]. However, no difference in tear SP concentration was found between the post-12-month LASIK group and the control group [244]. Gao et al. [241] found that tear SP concentrations were inversely associated with corneal nerve fiber density; in particular, increased nerve density was correlated with lower tear SP levels. Studies on the changes in CGRP levels following LASIK have shown inconsistent results. Chao et al. reported that there was no significant increase in CGRP for up to 3 months after surgery in a prospective longitudinal cohort study [243], whereas in another cross-sectional study, the tear CGRP concentrations 12 months after surgery were significantly higher than those of normal subjects [244]. Mertaniemi et al. [242] investigated the effects of PRK on CGRP in a prospective study and found that, compared with the preoperative values, the amount of CGRP in tears increased and peaked on postoperative day 2 and thereafter declined through day 7. It was postulated that the significant elevation of CGRP release on days 1–2 may be attributed to secretion from damaged corneal stromal nerves. In particular, there was no significant decrease in tear CGRP concentrations despite the hypersecretion of tears after surgery, suggesting a simultaneous increased secretion of CGRP into tears, possibly by the corneal sensory nerves [242]. In summary, the literature implies that the levels of SP and CGRP are altered after refractive surgery. Further studies are needed to better delineate the changes in these and other neuropeptides over time and to correlate them with the clinical outcomes of DED.

#### 4.2.3. Dry Eye Related to Wearing Contact Lens Wear and Neuropeptides

A variety of biophysical changes to the tear film and ocular surface by contact lens wear have been reported including tear film instability, increased tear evaporation rate, decreased tear film meniscus volume, decreased basal tear turnover rate, increased tear osmolarity, lipid layer change with poor wettability, and increased conjunctival hyperemia and epithelial damage [245,246]. Only a few studies have reported on the effect of contact lens wear on neuropeptides, and the results remain controversial. In a study comparing 20 contact lens wearers and 20 non-wearers, the levels of SP and CGRP in tears were not different between groups and there were no differences in nerve morphology or ocular surface sensitivity [247]. However, they found relationships between nerve density, the level of CGRP in tears, and corneal sensitivity. In a study comparing 30 symptomatic and 30 asymptomatic contact lens wearers, the level of SP in tears was significantly higher in the symptomatic contact lens wearers, and SP was suggested to be potential biomarker for contact lens discomfort [245]. Although the volume of the related studies is small and additional research is needed, the results thus far suggest the possibility that an increase in SP level in tears may be related to discomfort or dry eye caused by wearing contact lenses, not to wearing contact lenses itself.

## 5. Limitations of the Research Performed to Date: What Is Missing?

Reports on the role of neuropeptides in various ocular diseases have recently increased. For DED, several studies also have been reported showing the role of various neuropeptides and neurogenic inflammation in the pathogenesis of DED recently. However, there are still many incomplete points for a complete understanding of their role. The most important limitations of recent results are the lack of clinical evidence and the inconsistency of the results reported by clinical and preclinical studies. As for SP, which has been widely studied recently using a mouse DED model, no significant difference in tear levels was found between the DED and control group in the clinical study [233]. This result raises concerns about the applicability of the results of preclinical studies, which showed clear improvements in the clinical signs of DED by antagonizing NK1R [218,220,221] to humans. Considering that the patients included in the aforementioned clinical study included those with relatively advanced DED (one-half of the DED patients had Sjögren’s syndrome and ocular cicatricial pemphigoid), an increase in SP level in tears may have been masked by the decreased corneal nerve density in the patients with advanced DED [47,49,50] and by subsequently decreased SP. Interestingly, there is a report that cyclosporine A, which has been clinically confirmed as a therapeutic agent for DED, inhibits binding of SP to NK1R, thereby suppressing SP-induced IL-6 secretion [248]. Although there is still no clear evidence that the inhibitory effect of cyclosporine A on the SP/NK1R system contributes the treatment effect in DED clinically, the fact that IL-6, which plays an important role in the pathogenesis of DED, can be inhibited through this mechanism suggests a possible contribution of inhibition of the SP/NK1R system to the treatment of DED. Further studies are needed to determine the contribution of cyclosporin A’s ability to antagonize NK1R among the various functions of cyclosporin A for the treatment of DED.

Although the mechanism related to SP, a representative proinflammatory neuropeptide, has been relatively extensively studied in relation to ocular surface diseases such as DED, the function of CGRP, which is known to be more prevalent in corneal nerves, is still lacking. CGRP may function to convert Th1 responses to Th2 responses on the ocular surface, which would potentially inhibit DED induction in response to hyperosmolar stress. NPY and VIP, which are known to be secreted mainly by the sympathetic and parasympathetic nerves, are not extensively distributed in the cornea, but are widely distributed in the lacrimal gland. Studies on the effects of other minor neuropeptides, such as α-MSH and PACAP, on DED have been conducted sporadically, but additional studies are needed to establish a more concrete relationship between these neuropeptides and DED.

## 6. Future Directions

Research on the role of neuropeptides in the pathogenesis of DED is still at a starting point, and more research is needed. Conceptual possibilities have been proposed for a long time, but experimental proofs have been delayed due to methodological difficulties. Although results from research in which these difficulties have been overcome have been recently published, it is expected that new methodological improvements will be needed for more in-depth research in the future, including genetically modified mice for in vivo study and diverse coculture techniques for studying neuronal cells and immune cells in vitro [242]. Since the immune response is a systemic response, where various immune processes work simultaneously, it is difficult to determine the contribution of each process. For example, in the preclinical studies related to SP [218,220,221], it is still not clear whether the source of the SP is the corneal nerves, as suggested by the concept of neurogenic inflammation, or recruited immune cells, as a result of induced inflammatory response. To analyze the effect of each process in an in vivo system, a genetically modified animal model may be the best tool for the evaluation of the separate effects [249]. Therefore, further study using genetically modified mice will be helpful to confirm the detailed mechanism of action of neuropeptides in DED pathogenesis. In this regard, it is encouraging that NK1R-knockout mice or Lyve-1^Cre^; VEGFR2^flox^ mice have been recently introduced to the study of ocular surface diseases [30,82]. Difficulty in performing in vitro studies using neuronal cell culture is another hurdle for the research that might reveal direct interactions between neuronal systems and immunologic systems. Recently, the trigeminal neuronal primary culture technique was used in some studies related to DED pathogenesis [218,219]. However, considering the possibility that the cells were in a slightly inflamed state due to trauma associated with the dissection performed to isolate the cells and their limited life span (typically days to weeks) [250,251], the in vitro primary neuronal cell culture model is limited for revealing the exact pathways in the pathogenesis of DED.

For clinical relevance, more clinical studies should be performed, starting with the changes in levels of neuropeptides in the tears of DED patients with more homogeneous DED profiles in a range of severity grades. In addition, expanding the scope of study on the pathogenic mechanism is needed to include diverse neuropeptides and diverse organs related to DED, including the cornea, conjunctiva, main and accessory lacrimal glands, meibomian glands, and neural networks.

The role of the ocular surface microbiome may also be an important factor to address in the future because the dominant Th17 cell response may be important in the pathogenesis of DED [32,33]. The concomitant microbiome condition accompanied by damage to the corneal nerve endings may be a determining factor for the future development of DED. Additionally, there have been reports indicating that cutaneous normal flora can be affected by neuropeptides secreted by sensory nerve endings, including SP and CGRP [252]. For autoimmune dry eye, such as that in Sjögren’s syndrome, the concept of a gut dysbiosis-ocular surface-lacrimal gland axis has been introduced [253,254,255]. There have also been reports on changes in the ocular microbiome resulting in increased Pseudomonas levels with contact lens use, increased staphylococcus levels in blepharitis and Stevens-Johnson syndrome, and increased Corynebacterium levels in DED and lax eyelid syndrome, as suggested by next-generation sequencing or whole-genome amplification [256,257]. Several studies have been directed to changes in the ocular microbiome in DED [257,258], but there has been no study on microbiome changes on the ocular surface in relation to neuropeptides.

Based on the information revealed by a murine animal model, it may be possible to prevent the exacerbation and progression to a more chronic form of DED by inhibiting the SP/NK1R system, especially in the early stages of DED. In addition to this candidate, clinical trials for possible therapeutic agents can be performed based on the concepts suggested in this review in the future. However, considering that neuropeptides have effects similar to a double-edged sword [5], the balance between their positive effects, such as suppressing the pathogenic mechanism of DED, and their negative effects, such as suppression of wound healing and innate immunity against infection, should be thoroughly evaluated before initiating clinical trials. 

Although research on the pathogenic role of neuropeptides in DED is still in its infancy, we believe that these concepts will contribute to broadening and deepening our understanding of the pathogenesis of DED and helping patients suffering from DED in the future.

## Figures and Tables

**Table 1:** Innervation of the organs related to dry eye disease.

Function	Nerve	Location of Nerve Cell Bodies	Main Distribution of Nerve	Minor Distribution of Nerve
Sensory nerves	Trigeminal nerve (CN V) Ophthalmic branch (CN V1) Maxillary branch (CN V2)	Trigeminal ganglion	Cornea (CN V1) [46] Conjunctiva (CN V1) [189] Eyelid (CN V1, V2) [190]	Lacrimal gland [191] Meibomian gland [192]
Parasympathetic nerves	Facial nerve (CN VII)	Pterygopalatine ganglion	Meibomian glands [192] Lacrimal gland [191] Conjunctiva [189]	Cornea [46]
Sympathetic nerves	Lateral horn to superior cervical ganglia	Superior cervical ganglion	Conjunctiva [189] Lacrimal gland [191]	Cornea [46]

CN = cranial nerve

**Table 2:** Distribution of nerve fibers expressing specific neuropeptides in the lacrimal functional unit according to species.

	Frequency of Nerve Fibers Expressing Each Neuropeptide According to Species			
	SP (+)	CGRP (+)	NPY (+)	VIP (+)
Cornea	Rabbit, cat, monkey: deep limbus/peripheral cornea > just beneath corneal epithelium, Rat: present in epithelium, stroma, and limbus	Rhesus monkey, human: around superficial limbal blood vessel/anterior stroma > epithelium, Rat: present in epithelium, stroma, and limbus	Rat, guinea pig, cat, rhesus monkey: around limbal blood vessel only, Rat: present in limbus and anterior stroma	Human: limited to limbal blood vessel (not in cornea proper), Rabbit: present *, Rat: present in limbus and anterior stroma
Ref.	[200,201,202]	[200,203]	[200,204]	[200,201,205]
Conjunctiva	Rat: present infrequently in epithelium, substantia propria close to vessels, and glands, Rabbit: present*	Rat: present moderately, mucocutaneous junction > epithelium/substantia propria/stroma in palpebral conjunctiva; present close to vessels and glands in bulbar conjunctiva	Rat: present moderately, scattered in parenchyma close to vessels and glands	Rat: present numerously, Human, mouse, rat, rabbit: present in epithelium and adjacent to goblet cells
Ref.	[201,206]	[206,207]	[206]	[201,206,208,209,210]
Lacrimal gland	Rat, guinea pig: duct system/blood vessel > interstitial space	Human, mouse: present rarely, Human: present in interlobular connective tissue, around blood vessels, and duct system	Human: present rarely, wall of arterioles > interlobular connective tissue	Human, mouse, rat, guinea pig: acini/around vessel > duct system
Ref.	[211]	[212,213]	[212]	[211,212,213]
Meibomian gland	Human: present sparsely, vessel > acini, Rhesus/cynomolgus monkeys, guinea pig: present in acini and vessel Rat: present sparsely, acini > vessel	Human: acini > vessel, rhesus/cynomolgus monkeys, rat, guinea pig: vessel > acini	Human, rhesus/cynomolgus monkeys, rat, guinea pig: present in vessel and acini	Human, rhesus/cynomolgus monkeys, rat: acini > vessels, Guinea pig: present in acini and vessels
Ref.	[192]	[192]	[192]	[192]

CGRP: calcitonin gene-related peptide, NPY: neuropeptide Y, Ref.: reference, SP: substance P, VIP: vasoactive intestinal peptide, * Histologic location was not specified.

**Table 3:** Preclinical studies to elucidate the role of neuropeptides/neuropeptides specific receptors in the pathogenesis of dry eye disease.

Related Neuropeptide	Species/Models	Administration Agent	Route of Administration	Highlights	Ref.
SP	Mice CEC dry-eye model	NK1R antagonist (1 μg/μL) (1) CP-99,994 (2) L-733,060	Topical	NK1R antagonist decreased the frequency of mature APCs in cornea and draining LNs, where APCs prime naïve CD4+ T cells to differentiate into Th17 cells.	[218]
SP	Mice CEC dry-eye model	NK1R antagonist (36 μg/100 μL) Spantide I	Intraperitoneal	NK1R antagonist ameliorated dry eye through restoration of Treg function and suppression of Th17-mediated responses.	[220]
SP	Mice CEC dry-eye model	NK1R antagonist (6.6 μg/5 μL) L-733,060	Subconjunctival	NK1R antagonism reduced clinical signs and corneal lymphangiogenesis in dry eye.	[221]
SP	Mice CEC dry-eye model	-	-	Cultivated trigeminal ganglion neurons harvested from dry eye-induced mice expressed and secreted higher levels of SP.	[219]
SP, CGRP	Mice (TSP-1 deficient) Sjögren’s type aqueous-deficient dry-eye model	-	-	CGRP substantially decreased but SP did not change in the cornea of older TSP-1 deficient mice. However, it has not been established how loss of TSP-1 alters the level of corneal neuropeptides.	[222]
α-MSH	Rat Scopolamine-induced dry-eye model	α-MSH (1, 10, 100 μg/mL)	Topical	α-MSH ameliorated ocular surface dysfunction and reduced proinflammatory cytokines by activating the PKA-ERK pathway in the scopolamine-induced dry eye rats.	[223]
PACAP, VIP	Mice (PACAP null) non-Sjogren’s type aqueous-deficient dry-eye model	PACAP38 (1 fM~1 nM) PACAP27 (0.1 nM) VIP (0.1 nmM or 10 nM) PACAP receptor antagonist (PACAP6-38) VIP receptor antagonist (VIP6-28)	Topical	PACAP eye drops stimulated tear secretion in the infraorbital lacrimal glands via the PAC_1_R/AC/cAMP/PKA/AQP5 cascade and suppressed dry eye signs.	[224]

AC: adenylate cyclase, α-MSH: alpha-melanocyte stimulating hormone, APC: antigen presenting cell, AQP5: aquaporin 5, cAMP: cyclic adenosine monophosphate, CEC: controlled environmental chamber, CGRP: calcitonin gene-related peptide, ERK: extracellular signal–regulated kinase, LN: lymph node, NK1R: neurokinin-1 receptor, PAC_1_R: PACAP receptor 1, PACAP: pituitary adenylate cyclase-activating polypeptide, PKA: protein kinase A, Ref.: reference, SP: substance P, Th17: IL-17-secreting CD4+ T cells, Treg: regulatory T cell, TSP-1: thrombospondin-1, VIP: vasoactive intestinal peptide.

## Data Availability

Not applicable.
